# Integration of a Digital Cognitive Assessment Tool in a Primary Care Setting

**DOI:** 10.1002/alz.089522

**Published:** 2025-01-09

**Authors:** Adam J. Doerr, Taylor A. Orwig, Mark N. Melkonyan, Robert P. Leung, John Showalter, Ali Jannati, Sage Purkey, Judy Mullavey, Stephen B. Erban, Bruce R. Weinstein, David D. McManus, Honghuang Lin

**Affiliations:** ^1^ UMass Chan Medical School, Worcester, MA USA; ^2^ Linus Health, Boston, MA USA; ^3^ Harvard Medical School, Boston, MA USA

## Abstract

**Background:**

Almost all primary care providers (PCPs) believe screening for mild cognitive impairment (MCI) and dementia in older patients is important. However, there are significant barriers in primary care, including low provider confidence in their assessment skills, time constraints, competing priorities, and poor financial incentives. Consequently, PCPs report conducting cognitive assessments for less than half of patients over 60 years of age. We evaluated the feasibility of integrating Linus Health’s Core Cognitive Evaluation (CCE), a tablet‐based digital cognitive assessment, into the workflow of a primary care practice at UMass Memorial Medical Center.

**Method:**

A convenience sample was recruited from six participating PCPs’ upcoming schedules. Eligibility criteria included age >65 years, ability to read, write, and speak English or Spanish, no prior diagnosis of cognitive impairment, and no known hearing or vision impairment. Patients were contacted for inclusion by phone one month prior to their scheduled visits and were instructed to arrive at their appointments one hour early. Participants were consented in person and were asked to perform the cognitive assessment on a tablet. PCPs reviewed the report during the visit and shared the results at their discretion. A report was uploaded to each participant’s REDCap record and linked to the encounter in the electronic health record.

**Result:**

The screened cohort consisted of 150 participants (mean age 74±6.7 years, 64% female). The CCE identified the results of 40 participants as borderline and 7 participants as indicative of cognitive impairment. Follow‐up actions taken by PCPs after positive screens included laboratory evaluations for reversible causes, referrals to neurology, and brain MRIs. As a result of taking the CCE, only 1% of patients reported feeling sad or depressed, while 5% reported feeling worried or anxious. More than two‐thirds of participants reported that taking the CCE was beneficial. All caregivers reported they would want to know if their loved ones’ brain health was declining. All PCPs reported they would continue using the CCE if made available following the study.

**Conclusion:**

Integration of the Linus CCE into a primary care setting was feasible and impacted PCPs’ clinical decision‐making, with high patient, caregiver, and provider satisfaction.

